# Development of Surface EMG for Gait Analysis and Rehabilitation of Hemiparetic Patients

**DOI:** 10.3390/s24185954

**Published:** 2024-09-13

**Authors:** Didier Pradon, Li Tong, Christos Chalitsios, Nicolas Roche

**Affiliations:** 1 Pôle Parasport University Hospital Raymond Poincaré, APHP, 92380 Garches, France; 2 U1179 Endicap, Versailles Saint Quentin University, 78000 Versailles, France; tong@k-invent.com (L.T.); roche.nicolas@aphp.fr (N.R.); 3 Kinvent, 34000 Montpellier, France; christos@kinvent.com; 4 Biomechanics Laboratory, Aristotle University of Thessaloniki, 57001 Thessaloniki, Greece; 5 Service d’Explorations Fonctionnelles, University Hospital Raymond Poincaré, APHP, 92380 Garches, France

**Keywords:** EMG, validation, stroke, gait

## Abstract

Background: The quantification of electromyographic activity using surface electrodes is invaluable for understanding gait disorders in patients with central nervous system lesions. We propose to evaluate a commercially available low-cost system compared to a reference system in participants with stroke-related movement disorders in functional situations. Methods: Three hemiparetic participants performed three functional tasks: two treadmill walks at different speeds and a sit-to-stand test. The vastus lateralis and gastrocnemius medialis muscles were equipped with two EMG sensors. The comparison between the two EMG systems was based on 883 identified cycles. Spearman’s correlation coefficients (SCs), linear correlation coefficients (LCCs), and cross-correlation coefficients (CCCs) were calculated. Results: The main results indicate good to very good similarity of the EMG signals collected from the two tested sEMG systems. In the comfortable-walking condition, an SC of 0.894 ± 0.091 and an LCC of 0.909 ± 0.094 were noted. In the fast-walking condition, an SC of 0.918 ± 0.064 and an LCC of 0.935 ± 0.056 were observed. For the 1 min sit-to-stand test, an SC of 0.880 ± 0.058 and an LCC of 0.881 ± 0.065 were noted. Conclusions: This study demonstrates good to very good similarity between the two sEMG systems, enabling the analysis of muscle activity during functional tasks.

## 1. Introduction

The quantification of electromyographic (EMG) activity using surface electrodes (sEMG) is invaluable for understanding gait disorders in patients with central nervous system lesions. Several objectives underpin the need to understand these gait disorders. Firstly, EMG quantification aids in the technological identification of muscles activated during walking. Numerous studies have detailed the contributions and activation levels of lower limb muscles [1,2]. For instance, Ref. [2] indicated the timing of different muscles’ activation during a normalized gait cycle. The authors observed that while timing may be similar, the intensity expressed as a percentage of maximal voluntary force differs between these muscles. Therefore, EMG activity quantification is particularly useful for identifying the nuances in muscle recruitment patterns. Quantifying these activation patterns by their temporal and/or intensity characteristics is also helpful to identify changes in muscle activation patterns, such as hyperactivity of certain muscles, co-contraction of agonist or antagonist muscle groups, or, to a lesser extent, muscle paresis.

This quantification, which identifies the timing and/or intensity of muscle activation, is essential for characterizing gait disorders and quantifying the impact of therapeutic interventions. The therapeutic management of gait disorders in patients with central nervous system lesions can be pharmacological, rehabilitative, surgical, or combined with walking aids [3,4,5,6]. For example, pharmacologically, Ref. [3] showed that the injection of botulinum toxin into the rectus femoris muscle reduced its abnormal activity observed during the swing phase via sEMG, leading to an improvement in peak knee flexion and gait quality. It is important to note that using botulinum toxin as a therapeutic solution to limit the injected muscle’s hyperactivity also reduces the muscle’s maximal force production and may temporarily weaken adjacent muscles [7]. In consequence, quantifying muscle activity via sEMG plays a clinical key role for understanding and managing gait disorders in patients with central nervous system lesions. This tool plays a crucial role in clinical evaluation, monitoring progress, and optimizing treatment strategies for these patients.

However, while sEMG quantification is easier to use than fine-wire EMG, it presents several limitations that must be considered. In clinical practice, in order to identify gait disorders resulting from abnormal muscle activities, following electrode placement recommendations for each superficial muscle is essential. No matter the patient-specific morphological or pathological characteristics, such as changes in soft tissue properties due to surgical interventions, the clinician is constrained to strictly adhere to these placement recommendations. In consequence, sometimes, these constraints may require the development of specific calculation codes from raw sEMG system data to combine scientific rigor with clinical utility.

Therefore, studies focusing on tool comparisons are as essential as studies characterizing the EMG activity of primary muscles activated during gait in patients with central nervous system lesions. Several studies have validated new sEMG systems by comparison with representative sEMG systems in their application domain, such as analyzing muscle activity in industrial settings [8,9] or, in our case, clinical settings [10,11]. Regarding the comparison of new systems with reference systems in industrial settings, Ref. [8] quantified parameters characterizing muscle fatigue to validate signal quality. More recently, Ref. [9] focused on similar applications with the same objective of fatigue quantification but using additional parameters. Concerning clinical applications, Ref. [10] compared an innovative system with a reference system using the maximal voluntary contraction of arm flexor and extensor muscles by quantifying the correlation level of the same variables between the two systems. Regarding lower limb muscle activity, Ref. [11] compared a low-cost system with a reference system using functional movements such as jump, squat, lunge, and knee extension, using validation indicators based on signal similarity.

Although many studies have been conducted over several years, we can categorize EMG sensor studies into three sections. The first category includes studies using EMGs to capture muscle activity to understand movements such as gait disorders [3]. This category also includes work on EMG signal processing methods and studies validating EMG sensors in specific contexts. The second category involves studies proposing new EMG signal processing and analysis methods to explore specific motor conditions such as fatigue [12], co-contraction [13], and muscle synergies [14]. The third category includes studies on the development or existence of new sensors, of which their use in specific fields, such as clinical settings, needs to be studied [11]. Our study fits into this dynamic, specifically evaluating new systems compared to reference systems by comparing signals recorded in functional situations. We proposed to evaluate a commercially available low-cost system compared to a reference system with participants with stroke-related movement disorders in functional situations such as comfortable and fast-paced walking, and conducted a sit-to-stand test.

## 2. Materials and Methods

### 2.1. Participants and Experimental Procedure

Three hemiparetic participants (two men, one woman) performed three functional tasks: two treadmill walks at different speeds and a sit-to-stand test. These participants undertook these motor tasks as part of the inclusion visit of a research protocol on the effect of repeated botulinum toxin injections [Committee for the protection of persons île-de-France 2015-A01671-48, NCT02699775].

The inclusion visits aimed to identify participants meeting the inclusion criteria, which, for this study, were hemiparetic patients post stroke (more than six months), capable of autonomous walking or with simple technical assistance, and with gait disorders associated with muscle hyperactivity.

Participants who agreed to the inclusion tests used a simple cane (two participants) or an AFO (one participant) for walking. Both aids were used only for outdoor mobility: on uneven terrain (sidewalks, slopes, gravel) and in unfamiliar or crowded places (shopping). The participants had an ischemic stroke more than 8 years ago, resulting in left hemiparesis for the woman and right and left hemiparesis for the two men. Each participant was more than three months post treatment for muscle hyperactivity with botulinum toxin injections and was not on any medication. 

During this visit, some participants were equipped with a second sEMG system certified as a medical device in accordance with European medical device regulations. These participants were accustomed to treadmill walking due to their weekly rehabilitative care at their medical facility.

The functional tasks were chosen arbitrarily but are representative of the rehabilitative care for this population. We selected two locomotor tasks on a treadmill lasting three minutes: the first at a self-determined comfortable speed and the second at a self-determined fast speed. We did not impose a common speed since the inclusion visit aimed to identify hyperactivity in one or more muscles engaged during walking and, for this study, we sought to compare signals recorded by two different systems on the same muscle. The sit-to-stand task corresponded to the one-minute sit-to-stand test, commonly used to evaluate the motor and physical abilities of various pathologies [15].

**Figure 1:**
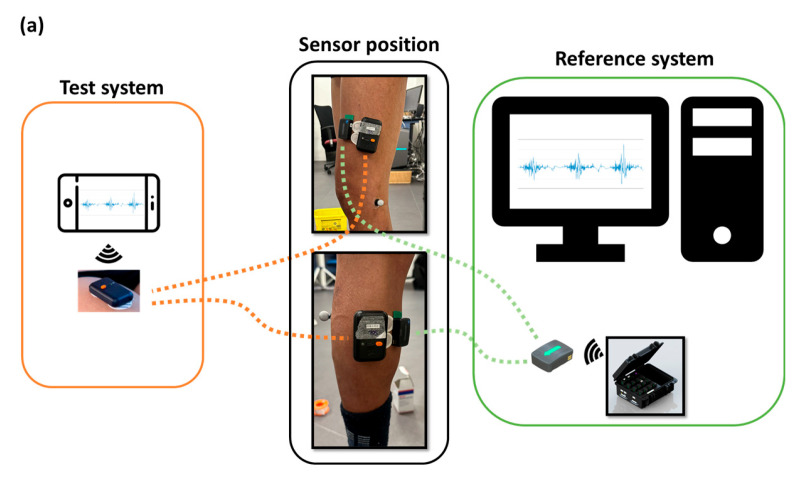

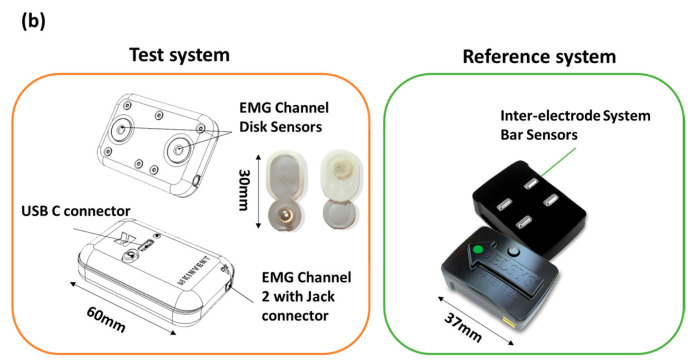
Block diagram describing (**a**) operation and use, (**b**) certain features and dimensions for the two systems: the Delsys system (Reference system) and Kmyo system (Test system).

Two muscles were equipped with two different sEMG systems (the Kinvent Kmyo system and the Trigno Avanti Delsys system detailed in the sections below). We equipped the vastus lateralis and gastrocnemius medialis muscles (Figure 1a). These muscles were intentionally chosen because they met two criteria: involvement in locomotor tasks and sufficient size to accommodate two sEMG sensors from different systems. The electrodes were positioned following the recommendations of the SENIAM project (Surface ElectroMyoGraphy for the Non-Invasive Assessment of Muscles, http://www.seniam.org/, accessed on 26 June 2024). For the vastus lateralis, electrodes need to be placed at 2/3 on the line from the anterior spina iliaca superior to the lateral side of the patella. For the gastrocnemius medialis, electrodes need to be placed on the most prominent bulge of the muscle.

### 2.2. Material: EMG System

The Kinvent Kmyo is a dual-channel wearable sEMG sensor. The first channel is tailored to straightforward application, enabling direct connection to self-adhesive pre gelled electrodes with 3.5 mm snap connectors. The second channel, designed for high-precision measurements, incorporates a three-electrode wired setup with a Right Leg Drive node for enhanced noise reduction. It operates at a maximum sampling frequency of 2000 Hz, with adjustable amplification gains, and utilizes a 24-bit Analog-to-Digital Converter (ADC) to ensure a signal resolution of 0.1 uV and baseline peak-to-peak noise below 10 uV. This study uses the integrated channel with 2 self-adhesive pre-gelled electrodes.

The Trigno Avanti Sensor integrates an sEMG sensor with an IMU within its compact design. This facilitates detailed motion and muscle activity analysis using a single device. The Trigno Avanti Sensor used a dry electrode fixed on the body using double-sided tape. The technical information of both systems is summarized in Table 1 and Figure 1b.

### 2.3. Data Analysis

Following the completion of all exercises and the recording of signals from both the Kmyo System and the Delsys System, we proceeded with a thorough data analysis. 

The recording of self-determined comfort and fast speed walking generated 361 and 443 cycles, respectively, for the two muscles. The one-minute sit-to-stand test recording yielded 59 cycles.

EMG signals are particularly prone to various types of noise and artifacts that can obscure the actual muscle activity. Sources of noise include electrical interference from power lines, motion artifacts due to electrode movement, and physiological noise such as other muscle activities or cardiac signals. Additionally, factors like electrode placement, skin impedance, and muscle fatigue can affect signal quality. In this study, it was challenging to shave and abrade the patients’ skin, so we only cleaned the skin with an alcohol swab for medical use, which also degreased the skin. Proper signal processing is crucial to remove these unwanted components and ensure accurate interpretation and reliable results [16,17,18].

Our analysis method involves a multi-step signal processing procedure and the assessment of performance indicators to compare the Kmyo System with the Delsys System. The 5 signal processing steps described in Section 2.3.1 are as follows [19,20,21]:

(1) Signal filtering to remove noise;
(2) Synchronization to align the data accurately;
(3) Rectification;
(4) Normalization to facilitate comparisons between different electrode sites and envelope calculation to smooth the signal amplitude over time;
(5) Data trimming to segment the gait cycles.

The validation indicators and size effect, described in Section 2.3.2 and Section 2.3.3, used to evaluate the similarity and relationship between the signals from the two systems include the following [17,22]:

(1) Spearman’s correlation;
(2) Linear correlation coefficient (LCC);
(3) Cross-correlation coefficient (CCC);
(4) Effect Size.

By implementing these steps, we aimed to achieve a comprehensive and reliable comparison of the Kmyo System’s performance against the Delsys System, ensuring the validity of our results for various research and clinical applications.

#### 2.3.1. Signal Processing

**Figure 2:**
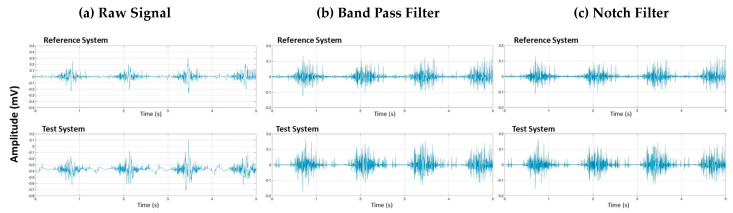
Vastus lateralis signal signal processing of raw data (**a**) after Band-pass filtering (**b**) and after Notch filtering (**c**) for two systems: the Delsys system (Reference system) and Kmyo system (Test system).

Signal processing involves several steps to prepare the raw EMG data for analysis. These steps include signal filtering, synchronization, rectification, normalization, envelope calculation, and data trimming (Figure 2).

##### Signal Filtering

Signal filtering is the process of removing noise and artifacts from the raw EMG data to enhance the quality and reliability of the signal. The purpose of filtering is to eliminate unwanted components, such as low-frequency movement artifacts and high-frequency noise, which can obscure the true muscle activity signals.

To achieve this, we applied a band-pass Butterworth filter with a frequency range of 40–400 Hz, which effectively removes low-frequency and high-frequency noise (Figure 2b). Additionally, we used a notch filter centered around 50 Hz to eliminate power line interference (Figure 2c). These filtering steps ensure that the remaining signal accurately represents the muscle activity by minimizing external and physiological noise sources.

##### Signal Synchronization

Signal synchronization is crucial to align the EMG data accurately for comparative analysis. Synchronization ensures that signals from different sensors are temporally aligned.

For accurate comparative analysis, the synchronization of the sEMG signals from four independent sensors was paramount. The Kmyo System and Delsys System feature inherent inter-system synchronization capabilities. Synchronization between these commercial systems was achieved using a Kmove sensor from Kinvent and the 3D motion analysis system. The Kmove, an IMU-based motion sensor, collects detailed kinematic data and wirelessly synchronizes with the Kmyo System. Simultaneously, the 3D motion analysis system is synchronized with the Delsys System. At the onset of recording, the experimenter’s finger equipped with a reflective marker tapped the Kmove sensor in order to generate a significant acceleration signal peak. This event’s timestamp marks the synchronization point for the Kmyo System’s sEMG signals. The finger marker’s trajectory, captured by the 3D motion analysis system, identifies the tap moment, allowing for the synchronization of the Delsys System’s recorded sEMG signals.

##### Signal Rectification and Normalization

**Figure 3:**
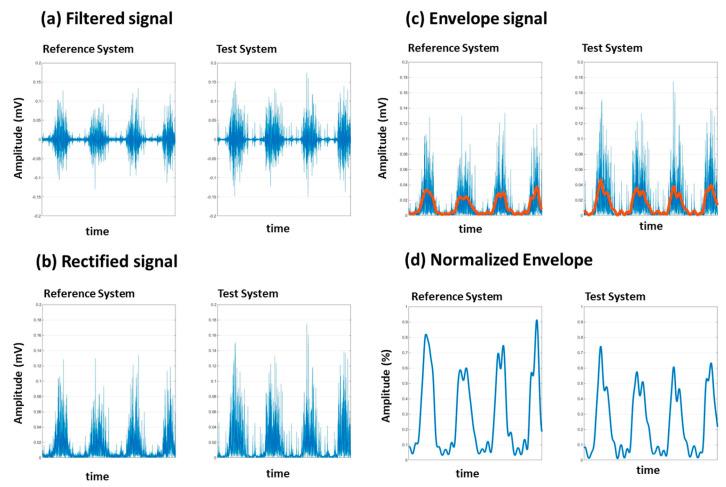
Vastus lateralis signal processing filtering (**a**), rectification (**b**), envelope (**c**) and normalization (**d**) for two systems: the Delsys system (Reference system) and Kmyo system (Test system).

Signal rectification and normalization are essential steps carried out to facilitate meaningful comparisons between EMG recordings from different electrode sites, muscles, or sessions. Rectification involves converting all negative values of the EMG signal to positive values, effectively creating a full-wave rectified signal (Figure 3b). This step ensures that the signal amplitude reflects the absolute value of muscle activity.

Normalization is necessary to facilitate comparisons between electrode sites on the same muscle, on two different muscles, or for documenting changes over days. In fact, normalization is a prerequisite for any comparative analysis of EMG signals.

Due to the physical limitations of our post-stroke subjects, performing maximum voluntary contraction (MVC) exercises was not feasible. Instead, we used the peak value recorded during each session for normalization (Figure 3d). This approach, validated in prior research, provides an effective alternative for populations where MVC is challenging to achieve. By normalizing to the session’s maximum value, we ensured that the variations in signal amplitude are attributed to muscle activity rather than extrinsic factors [23]. Recently [23], a study explored using movements other than maximal voluntary isometric contraction to normalize EMG signal amplitude during gait. The results suggest that using maximal amplitude identified in functional movements is a viable normalization alternative when participants have difficulty performing maximal voluntary contractions. Due to their impairments, our participants had difficulty performing maximal voluntary contractions, especially of the gastrocnemius muscles.

##### Envelope Calculation

Envelope calculation is used to derive the amplitude envelope of the EMG signal, providing a smooth representation of the signal’s amplitude over time (Figure 3c).

To calculate the envelope, we used a Butterworth low-pass filter with a cutoff frequency of 5 Hz. This filter was applied to the rectified EMG signal using a zero-phase forward and reverse digital filtering method to avoid phase distortion. The result is a smooth envelope that represents the signal’s amplitude modulation.

##### Data Trimming

Data trimming is the process of segmenting the EMG data into specific intervals that correspond to the gait cycles for easier comparison and analysis. The EMG data were recorded while the participants walked on a treadmill. Data from the Kmyo System, the Delsys System, and a 3D motion analysis system were simultaneously recorded. After synchronization, we used the 3D motion analysis system to visually inspect and manually identify heel strike events, marked by reflective markers placed on the heel of the participants. These heel strike events were used to cut the timeseries data into gait cycles, facilitating more straightforward and accurate comparison of the EMG data.

#### 2.3.2. Validation Indicators

The validation indicators used in this study assess the similarity and relationship between the signals from the Kmyo System and the Delsys System. These indicators are calculated using the normalized signal envelopes obtained from both systems. The data used for these calculations include the filtered, rectified, normalized, and segmented EMG signals. The indicators are computed for each gait cycle, providing a detailed comparison of the signal envelopes across cycles. These validation indicators include Spearman’s correlation, Linear correlation coefficient (LCC), and cross-correlation coefficient (CCC), each offering a different perspective on the relationship between the two sets of signals.

##### Spearman’s Correlation (SC)

Spearman’s correlation is a non-parametric measure that assesses the monotonic relationship between two variables. It evaluates how well the relationship between the signals from the Kmyo System and the Delsys System can be described using a monotonic function.

Spearman’s correlation coefficient ranges from −1 to 1. A value of 1 indicates a perfect positive monotonic relationship, −1 indicates a perfect negative monotonic relationship, and 0 indicates no monotonic relationship. This measure helps in understanding how similar the rank ordering of the signals from the two systems is.

##### Linear Correlation Coefficient (LCC)

The linear correlation coefficient (LCC), also known as Pearson’s correlation coefficient, is a measure of the linear relationship between two variables. It evaluates how well the data points fit on a straight line when plotted against each other.

LCC values range from −1 to 1. A value of 1 indicates a perfect positive linear relationship, −1 indicates a perfect negative linear relationship, and 0 indicates no linear relationship. High LCC values indicate that the signals from the Kmyo System are linearly related to those from the Delsys System, demonstrating the Kmyo System’s ability to capture muscle activity similarly to the Delsys System.

##### Cross-Correlation Coefficient (CCC)

The cross-correlation coefficient (CCC) measures the similarity of two signals as a function of the time lag applied to one of them. It is used to determine how one signal matches with another when shifted in time.

CCC values range from −1 to 1. A value of 1 indicates a perfect match with an optimal time-lag, −1 indicates a perfect inverse match, and 0 indicates no correlation. High CCC values indicate a strong time-dependent similarity between the signals from the Kmyo System and the Delsys System, highlighting the temporal alignment and consistency of the muscle activity captured by both systems.

#### 2.3.3. Effect Size

Effect size measures the magnitude of observed differences [24]. Cohen’s d effect size represents the difference between two means expressed in standard deviation units (pooled standard deviation). The authors also note that absolute effect size is useful when the studied variables have intrinsic significance. In our study, preserving the RMS value calculated from the EMG signal envelopes from the two EMG systems is relevant as it characterizes the signal quantity.

## 3. Results

In the experimental tasks, data were collected from three subjects performing three different tasks: walking at self-selected comfortable and fast speeds and the one-minute sit-to-stand test. The EMG signals were recorded from two muscles, the gastrocnemius medialis and the vastus lateralis. A total of 881 cycles were collected for the two muscles and three experimental conditions. For the walking at a self-selected comfortable-speed task, a total of 361 cycles were collected from both muscles. In the walking at a self-selected fast speed task, 443 cycles were collected. For the one-minute sit-to-stand task, 59 cycles were recorded from both muscles. Table 2 summarizes the validation indicators, including the cross-correlation coefficient (CCC), linear correlation coefficient (LCC), Spearman’s correlation (SC), and effect size.

Effect size analysis indicates a very small difference between our two EMG systems in the three conditions (comfortable, fast, and 1MSTS). We can consider that the number of recorded and used cycles validates the null hypothesis between our two systems.

**Figure 4:**
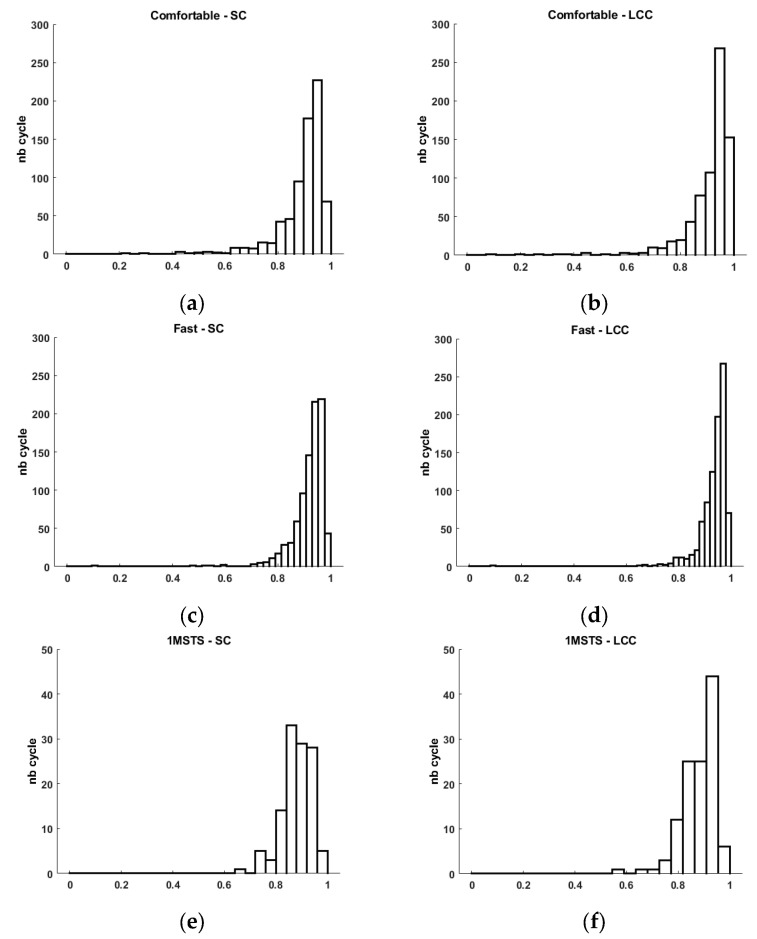
Histogram of Spearman’s correlation (SC) and linear correlation coefficient (LCC) for comfortable- (**a**,**b**) and fast-walking (**c**,**d**) tasks and 1MSTS (**e**,**f**).

The analysis of the EMG signals during the walking at a self-selected comfortable-walking speed task showed that the CCC values ranged from 0.864 to 0.997, with a mean of 0.975 and a standard deviation of 0.017. The LCC values ranged from 0.088 to 0.991, with a mean of 0.909 and a standard deviation of 0.094. Spearman’s correlation values ranged from 0.232 to 0.990, with a mean of 0.894 and a standard deviation of 0.091 (Table 2 and Figure 4).

For the walking at a self-selected fast speed task, the CCC values ranged from 0.876 to 0.997, with a mean of 0.978 and a standard deviation of 0.014. The LCC values ranged from 0.092 to 0.992, with a mean of 0.935 and a standard deviation of 0.056. Spearman’s correlation values ranged from 0.095 to 0.991, with a mean of 0.918 and a standard deviation of 0.064 (Table 2 and Figure 4).

In the one-minute sit-to-stand task, the CCC values ranged from 0.914 to 0.990, with a mean of 0.965 and a standard deviation of 0.018. The LCC values ranged from 0.576 to 0.973, with a mean of 0.881 and a standard deviation of 0.065. Spearman’s correlation values ranged from 0.649 to 0.966, with a mean of 0.880 and a standard deviation of 0.058 (Table 2 and Figure 4). 

## 4. Discussion

This study aimed to validate a low-cost sEMG system against a reference sEMG system to enable the analysis of surface muscle electromyographic activity during locomotor or functional tasks in patients with central nervous system lesions causing movement disorders. To achieve this goal, we compared the output signals of the two sEMG systems. Three chronic stroke patients with movement disorders performed two walking conditions and a functional task. In clinical evaluation activities of motor capabilities or difficulties, the quantification of muscle activities is necessary. However, although the indirect quantification of the force produced by the analyzed muscle is possible from a signal obtained under a condition where the person develops maximal voluntary muscle force, it remains difficult for patients with a central nervous lesion. Indeed, [25] indicates that for hemiparetic patients, following a central nervous system lesion of traumatic or vascular origin, a disturbance of motor control is observed [25]. This disturbance of motor control is a result of damage to the corticospinal pathways, also known as pyramidal syndrome, which corresponds to a set of neurological manifestations affecting voluntary motor control [25]. Thus, it is difficult to normalize the EMG signal collected for the analyzed muscles during locomotor tasks by performing a movement where the patient must voluntarily develop maximal force against resistance. However, to compare multiple tasks performed by the same patient, several studies have proposed using other situations than maximal voluntary isometric contraction, such as a movement at constant angular velocity or the studied motor task [26,27]. Given the population of our study, we integrated these works into our signal analysis steps. Additionally, in clinical evaluation activities of motor capabilities or difficulties, the quantification of temporal parameters of muscle contraction is pertinent information. Numerous studies have highlighted an association of movement disorders not by the amount of muscle force developed but by the timing of muscle activity during the performed motor task. For example, Ref. [3] indicates that the knee flexion deficit during the swing phase observed in hemiparetic patients is concomitant with the EMG activity of the rectus femoris muscle in the middle of this phase. These authors attribute the activity of this muscle to the fact that its contraction at this moment in the gait cycle limits knee flexion since the rectus femoris has a knee extension action. Similarly, Ref. [6] implicates the muscle activity of the triceps surae at the end of the swing phase as a potential cause of the plantar flexion strike, or the absence of EMG activity of the tibialis anterior at the end of the swing phase as a cause of the lack of ankle dorsiflexion [6].

For all these motor situations, the clinician needs to identify these muscle contractions. Therefore, the use of an sEMG system must be able to meet the constraints related to medical device regulations and enable this clinical analysis. This is why we chose the same similarity comparison criteria proposed by the authors of [11]. These authors proposed several indicators, including the Spearman coefficient, the linear correlation coefficient, and the cross-correlation coefficient. The cross-correlation coefficient is commonly used for qualitative EMG signal analysis in cyclic movements. Ref. [28] used this variable to analyze reproducibility between each gait recording in healthy subjects and between multiple sessions and examiners (electrode placement). For these authors, a CCC value >0.90 was an excellent score. 

The different gait or sit-to-stand cycles performed by the three participants on the two analyzed muscles allowed us to test the similarity of over 800 cycles. We specifically analyzed the similarity of 443 cycles for the comfortable-walking condition, 361 cycles at a fast pace, and 59 cycles for the 1MSTS test. For these different conditions and these three comparison criteria, our results indicate that the low-cost commercial sEMG has good to very good similarity with the reference sEMG. 

Firstly, in agreement with the work in [11], the cross-correlation coefficient (CCC) indirectly indicates the quality of post-processing synchronization as we note a nearly zero time lag. The calculated CCCs for comfortable walking, fast walking, and 1MSTS are, respectively, 0.975 ± 0.017, 0.978 ± 0.014, and 0.965 ± 0.018 (Table 2). Obtaining values of 1 is impossible due to the propagation of the electrical signal during muscle contraction. Although close, the EMG sensors are not exactly in front of the same muscle area. Thus, during muscle contraction, the electrical signal propagates, at a different latency, to different muscle localization [29]. This physiological constraint must be estimated, particularly by the cross-correlation coefficient, so that the lag does not impact the similarity calculation by other indicators (Spearman coefficient, linear correlation coefficient). Consequently, the closer the value is to 1, the more the interpretation of values obtained with other indicators will be related to the quality of the sEMG system.

For the comfortable-walking condition, we observe an average Spearman correlation coefficient of 0.894 ± 0.091. This result is consistent with the results obtained in [11]. A more in-depth analysis indicates that some cycles present a positive but weak similarity (SC min 0.232), while other cycles have very strong similarity (SC max 0.990). Graphically, we observe that the distribution of cycles with high similarity is much more significant than those with low similarity (Figure 4). This graphical observation also helps to understand the low calculated standard deviation. This analysis is similar for the linear correlation coefficient. Indeed, we quantify an average LCC of 0.909 ± 0.094 with a minimum value of 0.088 and a maximum of 0.991.

For the fast-walking condition, we observe an average Spearman correlation coefficient of 0.918 ± 0.064. A more in-depth analysis indicates that some cycles present a positive but very weak similarity (SC min 0.095), while other cycles have very strong similarity (SC max 0.991). As with comfortable walking, we observe graphically that the distribution of cycles with high similarity is much more significant than those with low similarity (Figure 4). This graphical observation also helps to understand the low calculated standard deviation. This analysis is similar for the linear correlation coefficient. Indeed, we quantified an average LCC of 0.935 ± 0.056 with a minimum value of 0.092 and a maximum of 0.992. For an industrial user, it is certain that obtaining the highest possible values is preferable, but it is also necessary to provide explanations when some cycles have positive but weak correlation coefficients. We believe that these few cycles with low similarity may result from motion artifacts as described by the authors of [30]. These authors detail all the best practices necessary to obtain a high-quality EMG signals. Despite adhering to these recommendations, such as cleaning the skin before placing the sEMG electrodes, no recorded signal can be entirely perfect regardless of the commercialized EMG system. Thus, cycles with low similarity may correspond to motion artifacts contained in the signal of the low-cost system as well as the reference system. In clinical activities aimed at studying movement disorders, particularly those associated with disturbances in muscle activity, the possibility that the recorded signal from a commercialized system may be subject to artifacts is a guarantee of quality, as surprising as it may seem. Thanks to numerous works that have long described the elements affecting EMG signal quality related to recording, processing, and sEMG electronics, we can identify these artifacts during analysis and interpretation [16,29,30]. Thus, in the context of analyzing movement disorders, especially for patients with central nervous system lesions, identifying these clinically considered disturbing muscle activities is essential. Numerous studies incriminate several muscles for different movement disorders, facilitating identification during the analysis of EMG signals obtained during clinical examination with the patient [3,6]. However, although these works greatly assist the clinician in understanding the specific movement disorders of their patient, interpretation remains specific for each patient to allow the clinician to consciously choose the therapeutic options they will propose. This clinical reality requires trust in the analysis equipment. This trust relies on regulatory and scientific validity, as well as the system’s sensitivity and, consequently, its exposure to motion artifacts and other sources of disturbance, which are increasingly and better reduced. On this point, we agree with the validation indicators used by the authors of [11], as they quantified the level of similarity between two signals but also, by indicating the minimum similarity values, allow us to appreciate the system’s sensitivity to motion artifacts, among others.

These disturbances were only slightly present in the 1MSTS condition. Indeed, we obtained a minimum SC of 0.649 and a minimum LCC of 0.576. For the rest, all the validation indicators show good similarity, as we quantified an average SC of 0.880 ± 0.058 and an average LCC of 0.881 ± 0.065. These results are higher than those obtained by the authors of [9]. These authors also used Spearman’s coefficient to compare a low-cost prototype system with a reference system similar to ours. It is important to note that the authors did not perform the same locomotor movements. They asked participants to perform different conditions lasting 120 s, including a Frankenstein walk, a sidewalk, a wall sit, and squats. The latter movement is similar to our 1MSTS. They achieved good results, with the SC ranging from 0.670 to 0.710. In our study, we used a commercially available low-cost system. Results with a commercial system, low-cost or not, should be superior to those of a prototype. This is what we observe (Table 2). However, our values, while considered good, are lower in the two walking conditions. The sit-to-stand movement involves a greater range of hip and knee flexion/extension movements than walking. We can think that the skin motion artifact may be more significant in this condition than in the other two and thus impacts the signal quality. This ultimately can increase the number of cycles with good rather than very good similarity (Figure 4).

The comparison of new sEMG systems with reference systems in our user community of clinicians is essential. Whether we have usage objectives for guiding therapeutic choices or in answering research questions, we cannot solely rely on compliance with medical device regulations. Our work contributes to the scientific community’s evaluation dynamics of these new devices. Thus, in line with the validation indicators proposed by [11], we observed good to very good similarity of EMG signals collected between low-cost commercialized sEMG systems and reference systems for analyzing movement disorders in patients with central nervous system lesions.

## 5. Conclusions

The comparison of new surface electromyography (sEMG) systems with reference systems within our community of clinical users is essential. This study aimed to compare two commercial sEMG systems, one low-cost and the other a reference standard. A total of 863 cycles from three functional conditions involving two lower limb muscles in three stroke patients were analyzed. The similarity analysis of sEMG signals using validation indicators such as Spearman correlation coefficients, linear correlation coefficients, and cross-correlation coefficients indicates good to very good similarity between the two sEMG systems. This work contributes to the ongoing evaluation of new medical devices by the scientific community.

## Figures and Tables

**Table 1:** Technical information for Kmyo (Kinvent) and Trigno Avanti systems (Delsys).

	Trigno Avanti Sensor	Kinvent Kmyo
**Dimensions**	27 × 37 × 13 mm	64 × 40 × 16 mm
**Weight**	14 g	30 g
**Battery life**	8 h	12 h
**Input differential range**	11 mV/22 mV	186 mV
**Sensor resolution**	16 bits	24 bits
**EMG baseline noise (typical)**	0.75 uV	<1.0 uV
**Number of channels**	1	2
**Sampling rate (max)**	4370 Hz	2000 Hz
**Synchronization accuracy**	<1 sampling period	<1 sampling period

**Table 2:** Values of the cross-correlation coefficient (CCC), Spearman’s correlation (SC), linear correlation coefficient (LCC) and effect size, for comfortable- and fast-walking tasks and one-minute sit-to-stand test (1MSTS).

		Tasks		
		Comfortable Walk	Fast Walk	1MSTS
**CCC**	**Min**	0.864	0.876	0.914
	**Max**	0.997	0.997	0.990
	**Mean**	0.975	0.978	0.965
	**Std**	0.017	0.014	0.018
**SC**	**Min**	0.232	0.095	0.649
	**Max**	0.990	0.991	0.966
	**Mean**	0.894	0.918	0.880
	**Std**	0.091	0.064	0.058
**LCC**	**Min**	0.088	0.092	0.576
	**Max**	0.991	0.992	0.973
	**Mean**	0.909	0.935	0.881
	**Std**	0.094	0.056	0.065
**Effect Size**		0.0501	0.1142	0.0868

## Data Availability

The original contributions presented in the study are included in the article, further inquiries can be directed to the corresponding author.
